# Using the Theoretical Domains Framework to Identify Barriers to and Enablers of Patient Telemedicine Services Use in China: Qualitative Study

**DOI:** 10.2196/78457

**Published:** 2026-01-21

**Authors:** Ke Liu, Yuting Yang, Zixuan Song, Huixian Li, Yanli Lyu, Ke Zhang, Xinxia Wu, Zheng Hou, Yipei Wang

**Affiliations:** 1 Department of Medical Affairs, Peking University Third Hospital, Beijing, China; 2 Office of Internet Hospital, Peking University Third Hospital, Beijing, China; 3 Institute of Hospital Management, Peking University Third Hospital, North Huayuan Road 49, Beijing, 100191, China, 86 01082266191; 4 Department of Otolaryngology, Peking University Third Hospital, Beijing, China; 5 Department of Gynecology and Obstetrics, Peking University Third Hospital, Beijing, China

**Keywords:** telemedicine services, Theoretical Domains Framework, barriers, enablers, patients, qualitative study

## Abstract

**Background:**

Telemedicine has rapidly expanded worldwide due to its convenience and accessibility. In China, an increasing number of hospitals have begun offering telemedicine services; however, patient utilization remains relatively low. Limited research has examined patients’ behaviors during the process of adopting telemedicine services.

**Objective:**

This study aimed to identify barriers to and enablers for patients using telemedicine services and to formulate implementation strategies.

**Methods:**

We conducted semistructured qualitative interviews based on the Theoretical Domains Framework (TDF) to identify barriers and enablers to telemedicine utilization. Twenty-one patients who had used Peking University Third Hospital’s telemedicine services were included in the interviews. Data were analyzed using NVivo 12.0 with deductive thematic analysis guided by the TDF. Moreover, a group of experts was assembled to devise potential intervention strategies.

**Results:**

A total of 28 themes were identified, including 14 barriers and 14 enablers across 5 of the 14 TDF domains. The most frequently reported barriers were operational challenges, prolonged waiting periods from asynchronous communication, and doubts about therapeutic efficacy, whereas the most frequently mentioned enablers were the convenience of telemedicine, time conservation, and support from hospitals. On the basis of these factors, we devised 6 intervention strategies.

**Conclusions:**

This study demonstrated that patients’ utilization of telemedicine services was affected by several barriers and enablers, including system architecture and design, patient interactions using telemedicine, and external assistance. To enhance the utilization, these factors must be meticulously considered. This study also suggests strategies to enhance the utilization of telemedicine.

## Introduction

With the rapid advancement of information technology, telemedicine services have expanded globally [1,2]. In China, the provision of telemedicine services by medical institutions has increased significantly since the COVID-19 pandemic [3]. Telemedicine services have expanded patients’ health care options [4]. Telemedicine is thought to enhance the efficiency of health care services [5,6], decrease medical costs [7,8], improve patient satisfaction [9], and facilitate the equitable distribution of medical resources worldwide [10,11]. Despite the growing number of institutions providing telemedicine, patient utilization remains low. As of 2023, more than 3000 institutions had provided telemedicine services, delivering nearly 100 million virtual visits. Despite the seemingly large service volume, telemedicine accounts for only 1% of all visits [12], a proportion further validated by patient interviews [13]. Conversely, almost 20% of medical consultations in the United States were performed via telemedicine in 2020 [14]. The establishment of telemedicine services without corresponding utilization has resulted in a significant waste of financial and human resources.

To enhance telemedicine utilization, it is crucial to identify and understand the factors influencing patient engagement. Previous studies have identified demographic factors, such as age [15], co-residence with children [16], and education [17], but these factors are difficult to change. Consequently, researchers turned to behavioral frameworks, such as the unified theory of acceptance and use of technology (UTAUT) [18], the technology acceptance model (TAM) [19], and the theory of planned behavior [20]. These frameworks have helped identify factors, such as perceived usefulness [21], ease of use [22], and social influence [23], as critical precursors to user intention. However, these models primarily focus on reflective processes, namely, how cognitive evaluations lead to the intention to use. Therefore, these classical models offer less granularity regarding the contextual barriers, habitual behaviors, and skill gaps that prevent intention from translating into actual usage in real-world health care environments.

The Theoretical Domains Framework (TDF) addresses this gap not by discarding prior theories but by synthesizing them into a more granular, intervention-oriented structure rooted in the capability, opportunity, and motivation system [24]. Thus, adopting the TDF allows for a true integration of classical constructs: for instance, the “perceived ease of use” in TAM is no longer just a perception but is further defined in operational context as “capability” (ie, the knowledge and skills domains); “social influence” in the UTAUT and the theory of planned behavior is recontextualized within “opportunity” (ie, the social influences domain); and “perceived usefulness” aligns well with “motivation” (ie, the beliefs about capabilities and beliefs about consequences domains) [25].

Therefore, the TDF effectively extends the theoretical boundary beyond the cognitive focus of TAM and UTAUT. By incorporating less-explored domains, such as environmental context and resources, the TDF captures the contextual drivers of behaviors that traditional models often overlook [26,27]. Its comprehensiveness enables our research to move beyond a simple prediction of adoption intention. Instead, it provides a diagnostic structure to identify relevant barriers and enablers affecting telemedicine usage [28,29]. Adopting the TDF thus situates our work within the existing literature, bridging the well-established cognitive constructs of TAM and UTAUT with the implementation-focused depth required for designing interventions aimed at improving patient utilization of telemedicine services in China.

Building on this theoretical foundation, this study targets patients who have used telemedicine services in China, aiming to examine their behaviors and psychological aspects during the telemedicine process. It used semistructured qualitative interviews based on the TDF to ascertain barriers and enablers to telemedicine adoption. Our research may offer actionable evidence to support the refinement of telemedicine platforms and the development of patient-centered service models in future practice.

## Methods

### Study Design

We conducted the survey in Beijing, a leading Chinese metropolis in the development of telemedicine. This study focused on tertiary public hospitals, which deliver the majority of telemedicine services in China and are highly trusted and sought after by patients [30,31]. Peking University Third Hospital, a tertiary public hospital, serves as the governing institution of the Beijing Telemedicine Quality Control Center [32]. It ranks among the top 10 hospitals in Beijing in terms of telemedicine user volume, with patients coming from dozens of provinces nationwide over recent years. The hospital provides both synchronous and asynchronous telemedicine consultations, covering the 2 predominant service models in China. Its extensive online services span the entire care process, including prediagnosis, diagnosis, and postdiagnosis.

### Participants Recruitment

We used convenience sampling to select patients engaged in real-time video and asynchronous graphic consultations at Peking University Third Hospital. We accounted for patients’ varied geographic locations, disease classifications, and socioeconomic statuses to ensure that the sample accurately represented the telemedicine-using patient community. The inclusion criteria were as follows: (1) at least one prior use of telemedicine at Peking University Third Hospital within the past 6 months, (2) willingness to participate in the interview, and (3) adequate communication skills to engage in the interview. Previous literature suggests that thematic saturation in qualitative interview studies is generally achieved before reaching 21 interviews [33]. Accordingly, the intended sample size was 20 to 22 participants in the preliminary phase. When the data collection process yielded no new information about the enablers and barriers influencing access to telemedicine services, data saturation was reached [34].

### Interview Guide Development

We applied the TDF throughout the interview design, data collection, and analysis. The TDF guided the development of the interview guide, which included 1 to 3 questions for each of the 14 domains (eg, goals and intentions) [35]. For example, one of the questions was, “What do you know about telemedicine services?” Investigators with expertise in implementation science provided guidance to develop the interview guide. Moreover, we consulted with experts in hospital management, health policy management, and physicians to refine the outline. We conducted 2 preexperiments after the outline was initially finalized. The interview outline was refined slightly after the pretests, and the samples from the pretests were not included in the study due to slight changes. Table 1 presents the TDF domains and corresponding interview questions.

**Table 1.** Interview questions aligned with the Theoretical Domains Framework (TDF) domains.

TDF domains	Definition [26]	Definition in this study	Interview questions
Goals	Mental representations of outcomes or end states that an individual wants to achieve	Goals that patients hope to resolve using telemedicine	What problems can telemedicine help you solve?
Intention	A conscious decision to perform a behavior or resolve to act in a certain way	Patients’ willingness or plan to use telemedicine	How did you know about telemedicine, and why did you try it? How do you feel about it compared to the offline service?
Knowledge	An awareness of the existence of something	The patient’s understanding of telemedicine	What do you know about telemedicine services?
Skills	An ability or proficiency acquired through practice	The patient’s practical ability to operate telemedicine independently	Are you familiar with using telemedicine services? Can you independently use telemedicine for online consultations?
Beliefs about capabilities	Acceptance of the truth, reality, or validity about an ability, talent, or facility that a person can put to constructive use	Patients’ confidence in their ability to use telemedicine successfully	To what extent do you feel capable of using telemedicine? When you use telemedicine, is there someone to help you? If so, who and how?
Optimism	The confidence that things will happen for the best or that desired goals will be attained	Patients’ positive expectations about the benefits of telemedicine	Do you think telemedicine can benefit patients? Is it beneficial to you? Please provide some examples.
Memory, attention, and decision processes	The ability to retain information, focus selectively on aspects of the environment, and choose between 2 or more alternatives	Cognitive aspects influencing telemedicine patients’ memory, concentration, and decision-making	Are there aspects that complicate the utilization of telemedicine services? Have you faced any particular challenges?
Emotions	A complex reaction pattern, involving experiential, behavioral, and physiological elements, by which the individual attempts to deal with a personally significant matter or event	Patients’ emotional experiences when engaging with telemedicine	How do you feel when you use telemedicine services? How do you feel about telemedicine services compared to offline service?
Beliefs about consequences	Acceptance of the truth, reality, or validity of outcomes of a behavior in a given situation	Patients’ perceptions of the benefits and risks of telemedicine	What are the enablers of telemedicine services? What are the barriers to telemedicine services?
Reinforcement	Increasing the probability of a response by arranging a dependent relationship, or contingency, between the response and a given stimulus	Incentives or feedback that motivate patients to use telemedicine	What incentives can motivate you to use telemedicine services?
Social or professional role and identity	A coherent set of behaviors and displayed personal qualities of an individual in a social or work setting	Patients’ perception of their role in health management and its alignment with telemedicine	Have people around you (family members, friends, etc) used telemedicine services? What attitudes do they hold about telemedicine services? Does their perspective affect your decision regarding the visit?
Social influences	Those interpersonal processes that can cause individuals to change their thoughts, feelings, or behaviors	Impact of family, peers, and social norms on telemedicine use	Do social environment and policies affect your use of telemedicine services? How?
Environmental context and resources	Any circumstance of a person’s situation or environment that discourages or encourages the development of skills and abilities, independence, social competence, and adaptive behavior	External conditions and resources that facilitate or hinder telemedicine use	Do you believe that the social environment and policies will influence your utilization of telemedicine services? What is its impact? Does the availability of resources and support affect your use of telemedicine? How?
Behavioral regulation	Anything aimed at managing or changing objectively observed or measured actions	Patients’ strategies to ensure effective use of telemedicine	What changes are necessary for telemedicine services to succeed? Do you have any other suggestions to enhance telemedicine usage?

### Interview and Data Collection

The formal interview was performed via telephone and approved in January 2024. We secured the participants’ informed consent and subsequently audio recorded the interviews using the software’s exclusive features. Each interview lasted approximately 10 to 20 minutes. The interviews were conducted in Mandarin Chinese by the authors KL and YY, both of whom had professional training in qualitative interviewing and extensive experience in qualitative research (n=2, one male and one female, health management practitioners). Before each interview, the interviewer provided a brief self-introduction and overview of the interview content to establish rapport with the participant. Only 2 of the interviewers were present during the interview to create a conducive environment for communication. The recordings were transcribed into textual materials within 48 hours after the interviews, and the researcher recorded and sorted the data to ensure consistency. After each interview, participants were sent a prearranged text message, and their data were collected.

### Data Coding and Analysis

Interview transcripts were transcribed, organized, and imported into the software NVivo (version 12.0; QSR International Pty Ltd) for coding. We conducted a deductive thematic analysis guided by the TDF and followed 3 steps [36,37]. First, the 14 TDF domains served as an a priori coding framework. Second, the transcripts were reviewed in detail, with relevant content identified and coded into themes. Third, these themes were categorized as either barriers or enablers within the corresponding TDF domains. When a theme was relevant to more than 1 TDF domain, it was assigned to the domain judged to be more closely aligned. Two authors (KL and YY) independently coded the transcripts. Coding discrepancies were resolved through discussion with a third author (YW) until consensus was reached. The coded κ for each level was greater than 0.90. To prepare the results, we tabulated the themes within each TDF domain and tabulated more prominent themes into barriers and enablers separately. A detailed description of the coding process, including example excerpts and coding framework, was provided in Multimedia Appendix 1.

### Strategy Development

Following encoding and analysis, we assembled a multidisciplinary panel of hospital experts, including specialists in information technology, clinical practice, and hospital administration leadership. This collaborative endeavor integrated technical, clinical, and operational viewpoints via monthly interdepartmental meetings and methodically structured brainstorming sessions employing implementation science frameworks. Using the TDF model, the panel systematically formulated various intervention options aimed at addressing the identified barriers and enablers.

### Ethical Considerations

This study was approved by the Medical Science Research Ethics Committee of Peking University Third Hospital (IRB00006761-M2024029). Participants provided informed consent. We stored the survey data, which were strictly managed to remain confidential. We offered a compensation of 100 yuan (US $13.8) as a token of appreciation to all participants for their involvement in the study.

This study followed established reporting standards for qualitative research and was reported in accordance with the COREQ checklist (Checklist 1).

## Results

### Participants and Institutions

All interviews were conducted from January to March 2024, covering a duration of 3 months. Twenty-five telephone interviews were conducted, of which 21 were considered legitimate for analysis. Of the 21 interviewees, 13 (62%) were women, and 19 (91%) held a bachelor’s degree or higher. The majority (18/21, 85%) of the participants had full-time jobs. The mean interview duration was 13.7 (SD 5.7) minutes. All participants had used telemedicine services at least once, with a mean of 3.46 (SD 2.8) visits as specified in Table 2.

**Table 2.** Interview patient characteristics (N=21).

Characteristics	Participants, n (%)
Sex	
Male	8 (38)
Female	13 (62)
Age (y)	
<30	4 (19)
30‐35	5 (24)
36‐40	7 (33)
>40	5 (24)
Education	
Senior high school or below	2 (9)
College	10 (48)
Postgraduate degree or above	9 (43)
Employment status	
Full-time employment	18 (85)
Unemployed	1 (5)
Retired	1 (5)
Student	1 (5)
Home location	
Beijing	13 (62)
Outside of Beijing	8 (38)
Having a chronic disease	
Yes	3 (15)
No	18 (85)
Undergone surgery	
Yes	3 (15)
No	18 (85)
Medical insurance type	
Employee insurance	14 (67)
Publicly funded free medical care	6 (28)
Urban resident insurance	1 (5)
Number of uses	
1	8 (38)
2‐5	8 (38)
6‐10	5 (24)

### Barriers to and Enablers of Patient Use of Telemedicine Services

A total of 190 principal assertions were documented, covering 5 domains and 28 themes, including 14 barriers and 14 enablers. Tables3  4 display the themes within each domain and their frequencies.

**Table 3.** Barriers to use of telemedicine services (n=71).

TDF^a^ domains and themes	Frequency, n (%)
Environmental context and resources (n=33)	
The interaction of the app is poor	12 (17)
The functions of the telemedicine services are limited	8 (11)
The process of telemedicine services and treatment is not smooth enough	5 (7)
The operation is not intelligent enough and is rather cumbersome	4 (6)
The propagandizing is insufficient	3 (4)
The environment is too noisy	1 (1)
Memory, attention, and decisions process (n=23)	
Patients have to wait passively for a long time by asynchronous communication	10 (14)
The operation is too complicated for the patients	10 (14)
It is hard to find the functions that suit oneself	3 (4)
Beliefs about consequences (n=8)	
Doubts and distrust about the effectiveness of telemedicine services	7 (10)
The risk of privacy leakage and security issues	1 (1)
Skills (n=6)	
Mobile phone operation skill is poor	4 (6)
It is difficult to collect information online	2 (3)
Beliefs about capabilities (n=1)	
Patients have no confidence in telemedicine services	1 (1)

a TDF: Theoretical Domains Framework.

**Table 4.** Enablers to use of telemedicine services (n=119).

TDF^a^ domains and themes	Frequency, n (%)
Beliefs about consequences (n=67)	
The convenience of telemedicine services	36 (30)
Telemedicine services can save time	16 (13)
Telemedicine services can enhance appointment accessibility	10 (8)
Telemedicine services can reduce transportation costs	5 (4)
Environmental context and resources (n=37)	
The system is easy to operate	10 (8)
Hospitals or app can provide guidance	8 (7)
Stable and fast network	8 (7)
Provide prompt consultation services	4 (3)
There is a quiet and suitable environment	4 (3)
Has the function of prescribing medicine	3 (3)
Beliefs about capabilities (n=8)	
The patient has self-efficacy in digital health	5 (4)
The patient has successful experience in online diagnosis and treatment	3 (3)
Skills (n=7)	
The patient’s mobile phone operation skills are relatively good	5 (4)
The patient has the ability to collect information	2 (2)

a TDF: Theoretical Domains Framework.

The barriers to theoretical domains were referenced a total of 71 times. Specifically, “environmental context and resources” was referenced 33 times; “memory, attention, and decision processes” was referenced 23 times; “beliefs about consequences” was referenced 8 times; “skills” was referenced 6 times; and “beliefs about capabilities” was referenced once. In contrast, the enablers of theoretical domains were referenced a total of 119 times. “Beliefs about consequences” was referenced most frequently, occurring 71 times”; “environmental context and resources” was referenced 37 times; “beliefs about capabilities” was referenced 8 times; and “skills” was referenced 7 times.

### Barriers

#### Environmental Context and Resources

“Environmental context and resources” was the primary barrier. It primarily emphasized user-environment interaction and the functional categories of telemedicine services. On the one hand, user-environment interaction explicitly examined how the design attributes of app influence patient engagement. It encompassed interface usability and system intelligence. On the other hand, the limited variety of functions created barriers. If patients’ medical needs were not fully met, they did not use telemedicine services.

*The software design seems flawed, insufficient prompts and guidance mechanisms make navigation unnecessarily challenging.*

[LQ, male, 2-time user]

*The functions of online consultation are too limited. It would be great if they could be more diverse to meet more demands.*

[KJ, female, 8-time user]

#### Memory, Attention, and Decision Process

The patients’ memory, attention, and decision-making processes might cause barriers, including asynchronous communication and the complexity of the process. First, barriers arose from asynchronous communication between patients and physicians. Following patients’ inquiries, they frequently endured prolonged delays before receiving a response from the physician, which significantly impacted patients’ readiness to use telemedicine. Second, barriers emerged from the complexity of the process. The complexity of the process was referenced 8 times in the interview, exclusively by first-time users. Patients’ attention was limited, but too many steps and jumps caused patients to resist the use of telemedicine services.

*Sometimes we want to contact the physician proactively and leave a message there, but the physician can’t see it. We can only wait for the physician to contact us. This is too passive.*

[LJT, female, 2-time user]

*I searched endlessly for the payment page but couldn't find it. The design isn't user-friendly.*

[YY, female, 1-time user]

#### Beliefs About Consequences

“Beliefs about consequences” manifested as barriers from two primary concerns: skepticism about treatment effectiveness and fears of privacy infringements. Primarily, owing to inadequate communication during telemedicine consultations, patients doubted the efficacy of telemedicine services. In addition, telemedicine necessitated the accumulation of extensive patient data, which led to concerns regarding the potential breach of personal privacy.

*I believe that online medical consultations are not as reliable as in-person visits. In a physical setting, physicians can conduct thorough examinations, whereas online consultations rely solely on verbal descriptions.*

[WWH, male, 1-time user]

*I am genuinely concerned about the potential for my personal information or medical data to be leaked. Given the prevalence of telecom fraud, I prefer to go to hospital.*

[YYX, male, 3-time user]

#### Skills

“Skills” emerged as a critical barrier domain, primarily manifested through deficits in digital health literacy and device operation proficiency. During the interviews, numerous patients indicated that telemedicine presented specific challenges for the older adults.

*At our age, we're barely comfortable with basic smartphone functions like WeChat. Navigating these sophisticated medical platforms feels overwhelmingly complex.*

[ST, female, 3-time user]

### Enablers

#### Beliefs About Consequences

“Beliefs about consequences” was repeatedly highlighted as an enabler, reflecting patients’ recognition of the benefits and potential outcomes of telemedicine. First, nearly all polled patients indicated that convenience facilitated their utilization of telemedicine services, with several patients reiterating this point consistently. Second, time conservation emerged as a key enabler, as telemedicine significantly reduced time expenditure, particularly for younger patients. Third, improved appointment accessibility was identified as a crucial enabler. Patients were able to schedule follow-up tests and obtain medications via telemedicine services without concerns regarding registration.

*After we use it, we think it is really convenient, so we don’t have to go to the hospital anymore, and it is not easy for us to go there.*

[YY, female, 1-time user]

*I think using this* [telemedicine services] *can communicate with the physician, the effect is similar, and it is much easier than offline, no need to ask for leave or go to the hospital.*

[SST, female, 1-time user]

*I've found departments are readily available on the telemedicine services platform. That’s really helpful.*

[TXM, female, 7-time user]

#### Environmental Context and Resources

“Environmental context and resources” was identified as an enabler influencing patients’ use of telemedicine services. First, app usability emerged as an enabler, as patients typically perceived that simple operation enhanced their engagement with the telemedicine services. Second, support from the app or hospital proved essential, helping patients easily locate access to telemedicine services and substantially improving utilization. Finally, the comprehensiveness of platform functionalities was recognized as a crucial enabler, as an extensive range of telemedicine services facilitated patients’ adoption and use.

*Telemedicine services are straightforward. I like it.*

[WZ, male, 5-time user]

*The hotline service is particularly helpful. Whenever I encounter difficulties, their staff guides me through the process step-by-step.*

[LJT, female, 2-time user]

*We hope they'll keep expanding features so we don't have to make endless trips to the hospital anymore.*

[PY, male, 2-time user]

#### Beliefs About Capabilities

“Beliefs about capabilities” evolved as enablers, reflected in two aspects: self-efficacy and prior successful experience. On the one hand, self-efficacy in digital health was largely associated with usage frequency, as expressed by confident users. On the other hand, patients’ previous successful experiences influenced their utilization behaviors, as they drew upon earlier eHealth encounters.

*I’m generally good at figuring out tech stuff. People around me always ask me for advice when they need online medical help.*

[YY, female, 1-time user]

*Having used other hospitals' internet services, I assume this APP should work fairly similarly.*

[KJ, female, 8-time user]

#### Skills

“Skills” emerged as a crucial enabler in the use of telemedicine services. In particular, expertise in using mobile phones and information retrieval was frequently mentioned. Individuals with advanced mobile device skills were more inclined to use telemedicine services. In addition, proficiency in information-seeking behaviors was recognized as an enabler.

*I'm quite comfortable using smartphones daily, so navigating these platforms seems easy.*

[LLZ, male, 1-time user]

*I independently researched all relevant information about telemedicine services. Honestly, I’m pretty good at finding what I need.*

[YY, female, 1-time user]

### Interventions to Improve Patient Utilization of Telemedicine Services

On the basis of these findings, we designed intervention measures addressing barriers and enablers through expert group discussions (Table 5). We assessed the importance of every domain by determining the frequency of citations throughout all transcripts. The domains “beliefs about consequences” and “environmental context and resources” were the most frequently cited, with 75 and 70 mentions, respectively, accounting for 40% and 37% of the total citations.

**Table 5.** Evidence-based interventions to improve telemedicine services utilization (n=190).

TDF^a^ domains	Frequency, n (%)	Intervention	Implementation details
Beliefs about consequences	75 (39)	Enhance support for first-time users	Pilot postdischarge coaching in orthopedics Train family physicians to assist virtual visits
Environmental context and resources	70 (37)	Simplify app functions	Suspend development of complex features Optimize core functionalities Streamline the patient’s usage process
Memory, attention, and decision process	23 (12)	Enhance APP guidance and 24/7 support hotline	Add real-time operation prompts Enable automatic step progression Develop an AI^b^-powered patient assistant Provide step-by-step guidance Establish rapid complaint resolution Implement AI-assisted feedback tracking
Skills	13 (7)	Multichannel publicity	Distribute instructional videos via WeChat or social media Install interactive kiosks in clinics Create a physician’s promotional webpage
Beliefs about capabilities	9 (5)	Age-friendly adaptation	Launch large-text interface Create elderly-specific tutorials Integrate voice command features

a TDF: Theoretical Domains Framework.

b AI: artificial intelligence.

In the “beliefs about consequences” domain, convenience emerged as a key factor encouraging patients to use telemedicine. Enhancing the experiences of first-time users was considered essential. It was advisable to offer supplementary services for patients using telemedicine for the first time, thereby enhancing their experience of its convenience.

Furthermore, in the “environmental context and resources” domain, the complexity of using the patient application represented the most prominent barrier factor. It was suggested to simplify the patient interface and limit the proliferation of complex features, hoping to facilitate patients’ focus on critical functions to meet the needs of the majority.

## Discussion

### Principal Findings

This study conducts an in-depth exploration of the relatively underresearched area of telemedicine among Chinese patients. It is the first instance of using the TDF model for conducting interview-based surveys with patients regarding telemedicine, thereby enhancing the scientific rigor of the research. This study found that telemedicine services adoption is jointly shaped by 14 barriers and 14 enablers, including 5 of the 14 TDF domains. This study has found more behavioral and psychological influencing factors in patients compared to other studies. Barriers arose from operational complexity, concerns about privacy and efficacy, asynchronous communication, and so on. Enablers include high convenience and accessibility, simplified system functionalities, and external support. On the basis of identified barriers and enablers, we developed targeted intervention measures and prioritized them based on their relative significance as expressed by the interviewees. In conclusion, this study advances a comprehensive understanding of the determinants influencing this adoption and establishes a theoretical foundation for developing targeted interventions in telemedicine services.

### Positive Feedback Mechanisms

Our exploratory analysis of usage frequency indicates that barriers and enablers change alongside patients’ cumulative experiences with telemedicine services. First-time or infrequent users were more inclined to highlight challenges associated with accessing the platform and operating system, indicating the significant learning curve of initial usage. In contrast, frequent users recognized constraints in the scope and profundity of accessible system functionalities while concurrently emphasizing convenience and efficiency as primary enablers. This transition suggests a possible positive feedback loop: as patients become accustomed to the system, operational obstacles lessen, and the perceived advantages of telemedicine become more prominent, hence encouraging further utilization.

This corresponds with previous research [13,38]. Similarly, in the TAM, it is believed that “perceived usefulness” influences behavioral intention [39]. Our research delineates a self-perpetuating feedback loop: utilization of telemedicine services leads to increased satisfaction and reinforced intention to use. This phenomenon highlights the importance of the first usage experience in telemedicine service adoption. Similarly, the anchoring effect in initial interactions profoundly influences long-term commitment. Facilitating smooth navigation and practical convenience during initial interactions might create positive behavioral anchors, promoting regular use of online telemedicine services. Previous research indicates that Michigan Medicine (Ann Arbor) has established a system that incorporates template instructions and anticipatory advice, which can enhance the patient utilization process [40]. Consequently, we advocate for the deployment of multimodal guiding systems, including artificial intelligence–driven triage and a human-assisted hotline.

### Duality of Barriers and Enablers

Our study reveals that the utilization of telemedicine services is directionally modulated by coexisting enablers and barriers within the same theoretical domains. The 2 domains, “environmental context and resource” and “the beliefs about consequences,” involve many barriers and enablers. Within the identical structural domain, both barriers and enablers arose, and the correlation between the two was notably strong.

The initial point is the discrepancy between functional complexity and operational simplicity. Within the “environmental context and resource” domain, patients simultaneously demand operational simplicity and comprehensive functionalities. To address patients’ requirements of operational simplicity, we must reduce the challenges to assure accessibility. This aligns with the findings of our and other research from the viewpoint of physicians [25,41,42]. The complexity of system operation significantly affects both physicians and patients. However, excessive feature expansion paradoxically increases interface complexity, hindering core functionalities. This phenomenon aligns with intrinsic cognitive load theory [43]. In the development of telemedicine services, it is imperative to address this equilibrium. This is a critical aspect that we meticulously consider while devising potential intervention strategies.

The second aspect is the trade-off between convenience and privacy. The “beliefs about consequences” domain encompasses opposing factors: perceived convenience and privacy concerns. In the study, the convenience of telemedicine services is the most frequently mentioned enabler by patients. However, this depends on patients supplying comprehensive personal information, medical history, and privacy. This also results in an elevated danger of personal privacy breaches. Although the issue of privacy breaches is only mentioned once in this study, it is frequently cited as a barrier in many studies on the use of telemedicine services [44]. Patients tend to resist using telemedicine services due to the risk of privacy breaches [45]. This also results in a simultaneous presence of dependence on convenience and sensitivity to contradictions, leading to conflicting and hesitant feelings in patients during the usage process [46].

### The Mismatch Between the Demand and Usage Capacity

In skills, digital literacy is a significant influencing element. Numerous interviewers highlighted the concern of digital literacy for older adults, despite the interviewers in the study being youthful. Older patients, despite increasing demand resulting from mobility restrictions and multimorbidity, face barriers due to limited digital literacy and adaptability [47,48]. In contrast, younger groups with technical skills demonstrate reduced demand. This generational mismatch hinders the use of telemedicine services by patients.

In establishing intervention strategies for telemedicine services, it is essential to address the requirements and features of both generations, rather than focusing on only one group. The alteration of text size was frequently referenced during the interview. Consequently, we devised an app modification strategy for the older people, offering extensive assistance while preserving the core functionality. We have also developed the “family affection account” feature, which enables children to assist the older adults in completing telemedicine-related processes.

### Legal and Security Concerns

In research primarily focusing on Western populations, privacy concerns are often cited as a major barrier to adoption [41,49-51]. Yet, this barrier was not prominent in our study, with only one participant explicitly raising privacy-related concerns. We speculate on the possible reasons from both macro levels and micro levels.

At the macro level, the social discourse and privacy awareness in China and Western countries differ markedly. In Europe and the United States, privacy protection is widely discussed [52], whereas in China, discussions regarding medical data privacy are limited, and most patients have low awareness, leading to infrequent expression of privacy concerns [52,53].

At the micro level, Chinese patients typically exhibit a high degree of trust in health care institutions, largely due to their public and government-regulated nature [54], which may further reduce concerns about potential data misuse [55].

### Limitations of the Study

This study has 3 main limitations. First, the sample consisted only of individuals who had already used telemedicine and were recruited from a single tertiary public hospital in Beijing, which may limit the generalizability of the findings. Future studies should consider including more diverse populations. Second, using a deductive approach to map participants’ statements to TDF domains may result in inconsistencies across coders. To mitigate this inherent limitation of deductive analysis, we used double independent coding, with any discrepancies resolved through adjudication by a third coder. Given the high consistency of participant narratives regarding barriers and enablers, we believe any residual impact is minimal. Third, as this study represents the first paper in a planned series, its primary goal was to identify barriers to and enablers of telemedicine adoption, thereby laying the foundation for intervention development. Thus, this study did not assess the effectiveness of proposed interventions. In future work, we will incorporate considerations of feasibility and other factors to prioritize specific interventions and rigorously evaluate their effectiveness.

### Conclusions

This study offered vital insights by identifying barriers to and enablers of patients using telemedicine services based on the TDF model. These findings not only genuinely represent patients’ experiences but also reflect the behavioral and psychological factors in using telemedicine services. This study revealed barriers, including operational difficulties, extended waiting times, and doubts concerning therapeutic effectiveness. Simultaneously, the enablers included the convenience of telemedicine, time conservation, and support from hospitals. Considering these criteria, we developed intervention strategies to enhance patients’ access to telemedicine. This research offers a solid foundation for developing more targeted intervention strategies and holds substantial practical implications for improving the telemedicine experience for patients.

## Supplementary material

10.2196/78457 Multimedia Appendix 1 The coding process and results (excerpt).

10.2196/78457 Checklist 1 COREQ checklist.

## Abbreviations

mHealth: mobile health
TAM: technology acceptance model
TDF: Theoretical Domains Framework
UTAUT: unified theory of acceptance and use of technology
