# Supplementary material for: Using the Theoretical Domains Framework to Identify Barriers to and Enablers of Patient Telemedicine Services Use in China: Qualitative Study
Source: JMIR Hum Factors. 2026 Jan 21;13:e78457. doi: 10.2196/78457 (PMC12823017; doi:10.2196/78457)
Supplement: Multimedia Appendix 1 [file humanfactors-v13-e78457-s001.docx]

**Table 1. The coding process and results (excerpt).**

| TDF domains | Factor type  (n) | Themes | Illustrative quote  (participant information) | Rationale |
| --- | --- | --- | --- | --- |
| Beliefs about consequences  (n=75) | Barrier (n=8) | 1. Doubts and distrust about the effectiveness of telemedicine services | 1. I believe that online medical consultations are not as reliable as in-person visits. In a physical setting, physicians can conduct thorough examinations, whereas online consultations rely solely on verbal descriptions. (WWH, Male) | **Theme coding:** This quotation exemplifies patients' doubts concerning the efficacy of telemedicine services.  **Domain coding:** It represents anticipated adverse outcomes, corresponding to the beliefs about consequences domain. |
|  |  | 2. The risk of privacy leakage and security issues | 2. I am genuinely concerned about the potential for my personal information or medical data to be leaked. Given the prevalence of telecom fraud, I prefer to go to hospital. (YYX, Male) | **Theme coding:** The participant expresses concern over privacy and security risks.  **Domain coding:** These perceived risks influence attitudes toward telemedicine adoption. |
|  | Enabler (n=67) | 1. The convenience of telemedicine services | 1. After we use it, we think it is really convenient, so we don't have to go to the hospital anymore, and it is not easy for us to go here. (YY, Female) | **Theme coding:** Patients perceive telemedicine as convenient, reflecting a positive expected outcome.  **Domain coding:** Represents anticipated benefits, aligned with beliefs about consequences. |
|  |  | 2. Telemedicine services can save time | 2. I think using this (telemedicine services) can communicate with the physician, the effect is similar, and it is much easier than offline, no need to ask for leave or go to the hospital. (SST, Female) | **Theme coding:** Illustrates perceived time-saving benefits.  **Domain coding:** Reflects positive anticipated outcomes, aligned with beliefs about consequences. |
|  |  | 3. Telemedicine services can enhance appointment accessibility | 3. I've found departments are readily available on the telemedicine services platform. That's really helpful. (TXM, Female) | **Theme coding:** The participant highlights improved access to appointments through telemedicine.  **Domain coding:** This reflects a positive anticipated outcome, as enhanced accessibility can motivate telemedicine adoption, aligning with the beliefs about consequences domain. |
| Environmental context and resources (n=70) | Barrier (n=33) | 1.The interaction of the APP is poor | 1. The software design seems flawed, insufficient prompts and guidance mechanisms make navigation unnecessarily challenging. (LQ, Male) | **Theme coding:** The quote highlights system usability issues, which make telemedicine difficult to navigate.  **Domain coding:** Usability of the application represents an external factor influencing adoption, aligned with the environmental context and resources domain. |
|  |  | 2.The functions of the telemedicine services are limited | 2. The functions of online consultation are too limited. It would be great if they could be more diverse to meet more demands. (KJ, Female) | **Theme coding**: The quote highlights the insufficiency of functional features in telemedicine.  **Domain coding**: This aligns with the environmental context and resources domain, as limited functionality represents an environmental barrier to adoption. |
|  | Enabler (n=37) | 1. The system is easy to operate. | 1. Telemedicine services are straightforward. I like it. (WZ, Male) | **Theme coding:** This reflects the ease of system operation.  **Domain coding:** The ability to use the system effortlessly serves as a contextual enabler, aligning with the TDF domain Environmental Context and Resources. |
|  |  | 2. Hospitals or APP can provide guidance | 2. The hotline service is particularly helpful. Whenever I encounter difficulties, their staff guides me through the process step-by-step. (LJT, Female) | **Theme coding:** The quote describes support provided by hospitals or the application, which serves as an enabler.  **Domain coding:** The availability of guidance represents an external resource that facilitates telemedicine use, aligning with the environmental context and resources domain. |
|  |  | 3. Provide prompt consultation services | 3. We hope they'll keep expanding features so we don't have to make endless trips to the hospital anymore. (PY, Male) | **Theme coding:** The quote describes the variety of telemedicine functions is crucial. Patients adopt services when diverse features meet their needs.  **Domain coding:** We regard the number of functions of an APP as a significant aspect of environmental resources. |
| Memory, attention, and decision processes (n=23) | Barrier (n=23) | 1. Asynchronous communication | 1. Sometimes we want to contact the physician proactively and leave a message there, but the physician can't see it. We can only wait for the physician to contact us. This is too passive. *(LJT, Female)* | **Theme coding:** The quote illustrates delayed responses and lack of immediacy, corresponding to asynchronous communication.  **Domain coding:** Such delays increase cognitive load and impede patient decision-making. |
|  |  | 2. The operation is too complicated for the patients. | 2. I searched endlessly for the payment page but couldn't find it. The design isn't user-friendly, (YY, Female). | **Theme coding:** Illustrates complicated procedures and system operations.  **Domain coding:** Coded under Memory, Attention, and Decision Processes, as complex operations hinder memory retention and decision-making. |
| Skills (n=13) | Barrier (n=6) | Mobile phone operation skill is poor | 1. "At our age, we're barely comfortable with basic smartphone functions like WeChat. Navigating these sophisticated medical platforms feels overwhelmingly complex. (ST, Female)" | **Theme coding:** The quote highlights skill deficits that hinder telemedicine use.  **Domain coding:** Coded under Skills, as insufficient technical ability impedes effective utilization of telemedicine services. |
|  | Enabler (n=7) | 1. The patient's mobile phone operation skills are relatively good | 1. I'm quite comfortable using smartphones daily, so navigating these platforms seems easy. *(LLZ, Male)* | **Theme coding:** Highlights proficient operational skills that facilitate telemedicine use.  **Domain coding:** It emphasizes the importance of personal adequate technical ability. |
|  |  | 2. The patient has the ability to collect information | 1. I independently researched all relevant information about telemedicine services. Honestly, I'm pretty good at finding what I need. *(YY, Female)* | **Theme coding:** The quote reflects the patient's ability to search for and process information.  **Domain coding:** It emphasizes personal competence that facilitates effective telemedicine use. |
| Beliefs about capabilities (n=9) | Barrier (n=8) | 1. The patient has self-efficacy in digital health | 1.I'm generally good at figuring out tech stuff. People around me always ask me for advice when they need online medical help. (YY, Female) | **Theme coding:** The quote shows that the patient feels confident in handling digital health tasks, demonstrating clear self-efficacy.  **Domain coding:** That reflects the patient’s perception of their own ability to use telemedicine services. |
|  |  | 2. The patient has successful experience in online diagnosis and treatment | 2.Having used other hospitals' internet services, I assume this APP should work fairly similarly. (KJ, Female) | **Theme coding:** The quote reflects prior positive experiences with online services, supporting this theme.  **Domain coding:** Coded under beliefs about capabilities, as such experiences enhance confidence in using telemedicine. |
|  | Enabler (n=1) | 1.Patients is uncertain about their ability to efficiently utilize telemedicine services | 1.We don't think we can use the telemedicine services well. (LQ, Male) | **Why Theme:** The quote shows that the patient doubts their ability to use telemedicine effectively, indicating low confidence and limited self-efficacy.  **Why Domain:**It reflects the patient’s perception of their own competence in using telemedicine. |
